# NT-Pro-B-Type Natriuretic Peptide Levels in Infants with Failure to Thrive due to Caloric Deprivation

**DOI:** 10.1155/2010/983468

**Published:** 2010-05-04

**Authors:** L. B. Mänhardt, K. Norozi, C. Müller, C. Willaschek, B. Kostuch, R. Buchhorn

**Affiliations:** ^1^Department of Paediatrics, Caritas Krankenhaus, Uhlandstraße. 7, 97980 Bad Mergentheim, Germany; ^2^Department of Paediatrics, London Health Sciences Centre, London, ON, Canada N6A 5Wg; ^3^Department of Paediatric Cardiology and Intensive Care Medicine, Medical School Hannover, 30623 Hannover, Germany; ^4^Department of Laboratory Medicine, Caritas Krankenhaus, Uhlandstraße. 7, 97980 Bad Mergentheim, Germany

## Abstract

*Background.* Brain natriuretic peptide and its inactive fragment N-terminal pro-BNP (N-BNP) are reliable markers of ventricular dysfunction in adults and children. We analyzed the impact of nutritional state on N-BNP levels in infants with failure to thrive (FTT) and in infants with severe heart failure (HF). The purpose of this study was to compare N-BNP levels in infants with FTT with infants with severe HF and healthy controls. *Methods.* In a retrospective cohort study, we compared N-BNP levels from all consecutive infants with FTT and bodyweight below the tenth percentile (caloric deprivation (CD) group) to infants with severe HF. Reference values from infants between 2 and 12 month were taken from the literature and healthy infants. *Results.* Our results show that infants with FTT (*n* = 15) had significantly (*P* < .001) elevated N-BNP values compared with the healthy infants (*n* = 23), 530 (119–3150) pg/mL versus 115 (15–1121) pg/mL. N-BNP values in this CD group are comparable to the median value of infants with severe HF (*n* = 12) 673 (408–11310) pg/mL. There is no statistical significant difference in age. *Conclusion*. Nutritional state has an important impact on N-BNP levels in infants with FTT. We could show comparable levels of N-BNP in infants with FTT and infants with severe HF.

## 1. Introduction

Approximately 10% of children seen in primary care develop signs of failure to thrive (FTT). The clinical evaluation includes a thorough history and physical examination. Children who do not respond to treatment for inadequate caloric intake or have a suspected “organic” cause get further laboratory diagnosis to identify the 1% of cases with FTT that result from diagnosable disease. In addition to inadequate caloric intake and caloric absorption, excessive caloric consumption due to hyperthyroidism, malignancy, and chronic disease such as severe heart failure (HF) must be considered. 

Elevated N-Terminal pro brain natriuretic peptide (N-BNP) levels were found in children with severe HF due to congenital heart disease or dilated cardiomyopathy [1, 2]. From 2005 to 2008 we included N-BNP measurements in our laboratory program for infants with FTT in an attempt to use a laboratory test to identify infants who suffer from severe HF. Surprisingly we found significantly elevated N-BNP in infants with FTT without any evidence for cardiac disease. 

The purpose of this study was to compare N-BNP levels in infants with FTT with infants with severe HF and healthy controls from recently published N-BNP values.

## 2. Methods

**Figure 1:**
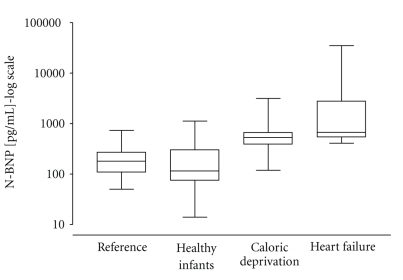
N-BNP values are illustrated as box and whiskers. The horizontal lines in the box plots reflect median value; the boxes reflect the 25th and 75th perecentile and the whiskers represent the maximum and minimum values: reference values from the literature [3], 23 healthy infants between 2 and 12 month, 15 infants with FTT, and 11 infants with severe HF. N-BNP values are on *y*-axis (log-scale). In comparison to the healthy infants the *P*-value of the CD group is high significant (*P* = .0002) and the *P*-value of the HF group in comparison to the CD group is not significant (*P* = .12).

The study design is a retrospective cohort study. Consecutive infants who were admitted to our hospital with FTT and bodyweight below the tenth percentile from 2005 to 2008 were included. Those infants with an organic cause of FTT like short bowel syndrome or endocrinologic disease were excluded. The remaining infants of the caloric deprivation (CD) group suffer from failure to thrive due to inadequate caloric intake and inappropriate type or volume of feeding. The infants with severe HF due to congenital heart disease were taken together in the “heart failure” group. History included the social standard of living, feeding behaviours, and family disorders. A whole body examination was performed. All infants received an echocardiography from a well-versed paediatric cardiologist. Normal N-BNP values shown in Figure 1 were taken from the literature [3]. For statistical analysis shown in Table 2, we received the original data for healthy infants from Norozi et al. [4] and Koch et al. [5]. These data were recently published in peer-reviewed journals. 

Peripheral venous blood samples were obtained from all participants, after a rest for at least 15 minutes. The subjects were in the supine position during blood sampling, which was typically done between 8:30 A.M. and 11:00 A.M. The blood samples were immediately placed on ice and subsequently centrifuged at 5000 rpm for 10 minutes. In all children N-BNP was determined using immunoassay (Elecsys 2010, Roche, Germany).

### 2.1. Exclusion Criteria

We excluded all infants with “organic” cause for FTT like short bowel syndrome or endocrinologic disease. Each patient had normal C-reactive protein values and therefore we could exclude significant inflammation.

N-BNP serum concentrations are markedly elevated in the first days of life in healthy neonates, probably due to circulatory changes in the perinatal period. All patients up to 31 days of life were excluded in both patient groups.

### 2.2. Statistical Analysis

Continuous variables were reported as median range and analyzed using nonparametric tests. For all parameters, a value of *P* < .05 was considered statistically significant. The Mann-Whitney test was used for comparison of N-BNP values of our patients and the original data from healthy infants published by Norozi et al. [4] and Koch et al. [5]. The data analyses were performed using Excel 2000 (Microsoft, USA) and Prism (GraphPad software Inc., USA). The study was approved by the local ethic committee.

## 3. Results

Demographic and height and weight characteristics for the three study groups are summarized in Table 1. 26 patients were included in our study, the CD group contained 15 patients, and the HF group contained 11 patients. 5 patients were excluded due to organic causes. As shown in Table 1 there is no significant statistical difference in age, bodyweight, body length, gestational age, and head circumference in the CD group and the HF group. Gestational age and birth weight were within normal limits. The CD group had a significantly lower postnatal weight gain 73 (0–147) g/week at home mostly caused by inadequate breast feeding at home, compared with the weight gain during hospital stay 225 (100–500) g/week. Further laboratory investigations show no significant difference for haemoglobin, white blood cells, ALT, and creatinine values in infants with HF compared to the infants with FTT. All these data were within the normal range. 

The HF group included 7 infants prior to cardiac surgery (ventricular septal defects or atrioventricular septal defects). Four infants suffered from severe HF more than 2 weeks after cardiac surgery (transposition of the great arteries, single ventricle). Seven infants with severe HF received a medical therapy with digoxin, diuretics, angiotensin converting enzyme inhibitors, and/or beta-blocker.

The laboratory investigations are shown in Table 2. For statistical analysis we compared the CD group with healthy infants and with the HF group. Our results show that infants with FTT had significantly elevated N-BNP values compared with the healthy infants 115 (15–1121) pg/mL versus 530 (119–3150) pg/mL, *P* < .001. N-BNP values in the CD group are not significantly different from values of infants with severe HF 673 pg/mL, (408 – 35000) pg/mL. 

As shown in Figure 1 N-BNP of our healthy infants group is in accordance with the reference values from the literature [3]. The median is nearly 4-fold higher in infants with FTT.

## 4. Discussion

Brain natriuretic peptide is secreted mainly by cardiac myocytes of the ventricles and to a lesser degree of the atria. Natriuretic peptides are released from the heart in response to pressure and volume overload [6]. However, several recent studies demonstrate an inverse relationship between body mass index and N-BNP levels were completed in adults with and without severe HF [7]. In this study we present significant N-BNP elevations in a group of 15 infants with CD. These infants only suffer from an inadequate caloric intake without any evidence for severe HF, congenital heart disease, and liver or kidney disease. HF and congenital heart disease in the CD group were excluded by echocardiography. There was no evidence of diastolic dysfunction on the echocardiograms, indicated by normal mitral valve inflow profiles. In our study the age of the remaining infants was in good accordance with the reference group of the literature [3]. The median N-BNP level of our infants with FTT 530 (119–3150) pg/mL) is comparable to children with Ross class III HF (744 pg/mL) [1]. Our heart failure group included only infants with severe HF with a very high N-BNP level of 408–35000 pg/mL. Interestingly, 58% of these infants with severe HF also suffer from FTT. These data are in good accordance with data from a pilot study regarding N-BNP in patients with HF due to ventricular septal defects showing that the perioperative change in N-BNP was most closely correlated with improved weight gain [2]. Also it is well known from adults with severe HF that cachectic patients have the highest N-BNP levels [8]. 

Although the reason for the relationship between body mass index and N-BNP is unknown, the increased concentration of the natriuretic peptide receptor-C clearance receptor on adipocyte cells has led some to postulate that increased clearance is the reason for lower N-BNP levels in obese patients [9]. However, evidence against this hypothesis comes from Das et al. [10], who found that N-BNP was correlated with greater lean mass but not greater fat mass. Cachexia in severe HF is associated with an increase in adiponectin concentration [11]. This may represent preservation of the physiological response to change in body fat but might also suggest that adiponectin plays a role in the pathogenesis of cachexia. The correlation between BNP and adiponectin also raises the possibility that the former might increase the secretion of the latter. In an experimental setting Tsukamoto could show that administration of BNP over 14 days in rats increased the plasma adiponectin concentration and concluded that BNP enhances adiponectin production in adipocytes, and BNP is a potential regulator of adiponectin production [12]. Ohara et al. found that the plasma adiponectin level increased along with an increase of plasma BNP in healthy subjects independently of other confounding factors and they concluded that adiponectin reflects cardiac function [13].

Our data are important for the interpretation of N-BNP levels in childhood. For example, the authors who published the reference values of N-BNP serum concentrations in healthy neonates and children did not give any information about the bodyweight of their study group [3]. This may be the reason for their high range of reference values (5–1121 pg/mL) in so-called healthy infants between 2 and 12 months.

 We urgently need reference values from infants with a well-documented normal nutritional state. Further studies may delineate whether measuring N-BNP levels are useful for the initial evaluation or ongoing management of patients with FTT due to either inadequate caloric intake or severe HF. 

The pathophysiological consequence of elevated natriuretic peptides in children with FTT with regard to volume regulation, blood pressure, and cardiovascular prognosis remains speculative. Our observation reveals new horizons for the explanation of well-known cardiovascular risk factors like the enhanced cardiovascular mortality in patients with low birth weight or FTT during infancy (Barker hypothesis) [14]. The link between low birth weight and FTT in infancy, catch-up growth in early childhood [15], and the higher risk of death from coronary heart disease in adults is important but yet not well understood. Longitudinal N-BNP measurements in these patient groups may help for a better pathophysiological understanding of Barkers hypothesis and risk stratification in infants with low birth weight and FFT. 

### 4.1. Study Limitation

A statistical problem is based upon the high range of N-BNP values in healthy infants especially in the first four weeks of life. These patients had to be excluded. Due to the nature of our retrospective study we were not able to measure adiponectin in our patients, which could be very helpful for interpretation of enhanced N-BNP, especially if there were a correlation between the level of adiponectin and N-BNP. Additional limitations of the study include the fact that there are a relatively small number of patients, and that data were obtained at a single time point without additional follow-up values.

## Figures and Tables

**Table 1:** Growth characteristics.

	Healthy infants	Caloric deprivation	Heart failure
*N*	23	15	11
Age (days)	166 (35–372)	160 (15–347)	90 (31–344)
Bodyweight < 3%		13	4
Bodyweight 3%–10%		2	2
Bodyweight > 10%		0	5

Bodyweight (g)	7600 (4800–10000)	4820 (2660–6700)	4350 (2755–8370)
Body length (cm)	69 (58–79)	62 (50–70)	59 (49–75)
Head circumference (cm)		40 (34–44.5)	38 (31–48)
Birth weight (g)		3100 (2130–3580)	3180 (1910–3670)
Gestational Age (w)		39 (36–41)	39 (34–42)

Values are given in median range, There is no significant statistical difference between “Caloric Deprivation” and “Heart Failure”.

**Table 2:** Laboratory results.

	Healthy infants	Caloric deprivation	Heart failure
*N*	23	15	11
NT-Pro-BNP (pg/mL)	115 (15–1121)	530 (119–3150)**	673 (408–35000)
Age (days)	166 (35–372)	160 (15–347)	90 (31–344)

	Normal Range		
HB (g/100mL)	11–15	11.3 (9.5–16.8)	12.1 (9.4–17.8)
WBC (1/mL)	5500–17500	10800 (6400–14200)	10000 (4500–22000)
CRP (mg/L)	<8	1.8 (0.00–2.36)	1.1 (0.00–2.12)
Creatinine (mg/dL)	0.04–0.68	0.23 (0.20–0.38)	0.36 (0.20–0.86)
Urea (mg/dL)	5.7–20.1	19 (9–28)	23.5 (10–70)
ALT (U/L)	0–45	30 (14–303)	24 (14–65)

Values are given in median range; Statistical Analysis using Mann Whitney Test between group “Healthy Infants” versus “Caloric Deprivation” and “Caloric Deprivation” versus “Heart Failure”. **P-value = .0002 (CD versus healthy infants).

## Abbreviations

ALT: Alanine aminotransferase
CD: Caloric deprivation
FTT: Failure to thrive
HF: Heat failure
N-BNP: N-Terminal pro brain natriuretic peptide.
